# Tuberculosis Antigen-Specific T-Cell Responses During the First 6 Months of Antiretroviral Treatment

**DOI:** 10.1093/infdis/jiz417

**Published:** 2019-08-17

**Authors:** Catherine Riou, Nishtha Jhilmeet, Molebogeng X Rangaka, Robert J Wilkinson, Katalin A Wilkinson

**Affiliations:** 1 Wellcome Center for Infectious Disease Research in Africa, University of Cape Town, South Africa; 2 Department of Medicine, University of Cape Town, South Africa; 3 Division of Medical Virology, Department of Pathology, University of Cape Town, South Africa; 4 Institute of Infectious Disease and Molecular Medicine, University of Cape Town, South Africa; 5 Department of Medicine, Imperial College London, London, United Kingdom; 6 The Francis Crick Institute, London, United Kingdom

**Keywords:** immune reconstitution, antiretroviral treatment, tuberculosis, protection, immune activation

## Abstract

The reconstitution of *Mycobacterium tuberculosis* antigen-specific CD4 T cells in a cohort of HIV-infected persons starting antiretroviral treatment (ART) in a high tuberculosis endemic area is described. Restoration of the antigen-specific CD4 T-cell subsets mirrored the overall CD4 T-cell compartment. Activation (assessed by HLA-DR expression) decreased during ART but remained elevated compared to HIV-uninfected persons. Despite known *M*. *tuberculosis* sensitization determined by interferon-γ release assay, 12/23 participants had no *M*. *tuberculosis*-specific CD4 T cells detectable by flow cytometry, combined with overall elevated T-cell activation and memory differentiation, suggesting heightened turnover. Our data suggest early ART initiation to maintain polyfunctional immune memory responses.

Human immunodeficiency virus (HIV-1) infection is the biggest risk factor for development of tuberculosis. Antiretroviral therapy (ART) reduces the risk of tuberculosis in HIV-1–coinfected persons. This therapy is associated with tuberculosis decline in sub-Saharan Africa between 2003 and 2016, with an estimated 1.88 million tuberculosis cases prevented [1]. However, the incidence of tuberculosis remains higher in HIV-1–infected persons despite ART (compared to those HIV-1 uninfected), regardless of the duration of ART or CD4 count [2]. This could be due to depletion of *Mycobacterium tuberculosis*-specific T cells early during HIV-1 infection [3], as well as impairment of function of these antigen-specific CD4 T cells [4]. Increased ART-mediated immunity correlates with expansion of early differentiated (central memory) T-cell responses overall [5–7]. However, *M*. *tuberculosis*-specific CD4 T-cell responses over the first 6 months of ART have not been studied in detail and data on the recovery dynamics of antigen-specific cellular immunity remain scarce. Here we compare the reconstitution of the whole CD4 T-cell compartment to that of the *M*. *tuberculosis* antigen-specific CD4 T-cell compartment, during the first 6 months of ART using flow cytometry, in a cohort of *M*. *tuberculosis*-sensitized HIV-infected persons starting ART, in an area with high prevalence of latent tuberculosis infection [8].

## METHODS

### Study Cohort

The University of Cape Town’s Faculty of Health Sciences Human Research Ethics Committee approved the study (REC: 245/2009) and written informed consent was obtained from all participants. Fifty HIV-1 infected persons were recruited at enrolment into the ART program from the Ubuntu Clinic in Khayelitsha, South Africa, as described [9]. Blood was collected longitudinally at baseline (day 0 of ART) and at 1, 3, and 6 months of ART. *M*. *tuberculosis* sensitization was determined by QuantiFERON Gold In-tube (QFT) assay and an in-house enzyme-linked immunospot assay (ELISPOT) as described and previously reported [9]. Remaining peripheral blood mononuclear cells (PBMCs) were stored for future use. Thus, 23 participants (median age 34 years, interquartile range [IQR], 29–39; 30% male) had stored PBMC available at all timepoints for the current analyses. All participants were ART naive at enrolment and *M*. *tuberculosis* sensitized as assessed by ELISPOT and QFT during the longitudinal follow-up [10]. None of these participants developed tuberculosis during the 6 months of longitudinal follow-up. An additional cohort of HIV-uninfected persons recruited from the same clinic were included as controls (n = 28; median age 28 years, IQR, 21–34; 50% male).

### Cell Stimulation and Flow Cytometry Analysis

Cryopreserved PBMC were thawed and rested in RPMI 1640 containing 10% heat-inactivated fetal calf serum prior to antigen stimulation. PBMC were stimulated using *M*. *tuberculosis* whole-cell lysate (10 μg/mL, strain H37Rv; BEI resources) for a total of 16 hours. Brefeldin A (10 μg/mL; Sigma) was added 3 hours after the onset of stimulation. After stimulation, cells were washed, stained with LIVE/DEAD Fixable Near-IR Stain (Invitrogen), and subsequently surface stained with the following antibodies: CD14-APC/Alexa Fluor 750 (Invitrogen) and CD19-APC/Alexa Fluor 750 (Invitrogen), CD4-FITC (Becton Dickinson), CD27-BV711 (Becton Dickinson), CD45RA-BV570 (Becton Dickinson), killer cell lectin-like receptor G1 (KLRG1)-PerCP/eFluor 710 (eBioscience), and HLA-DR–PE (Becton Dickinson). Cells were then fixed and permeabilized using Cytofix/Cytoperm buffer (Becton Dickinson) and stained with CD3-BV650 (Becton Dickinson), interleukin-2 (IL-2)–PE/Dazzle (Biolegend), tumor necrosis factor-α (TNF-α)–eFluor 450 (eBioscience), and interferon-γ (IFN-γ)–Alexa Fluor 700 (Becton Dickinson). Finally, cells were washed and fixed in 1% formaldehyde containing phosphate-buffered saline. Samples were acquired on a BD Fortessa flow cytometer and analyses were performed using FlowJo (V9.9.6, Treestar). A positive *M*. *tuberculosis* response was defined as at least twice the background (ie, without antigen stimulation). For cell surface phenotyping, only *M*. *tuberculosis* response with more than 20 events were considered. Cell polyfunctionality was analyzed using Pestle and Spice software. The gating strategy is presented in Supplementary Figure 1.

### Statistical Analyses

Statistical analyses were performed using GraphPad Prism (version 5.0). Nonparametric statistical tests were used for all comparisons. The Mann-Whitney U test and Wilcoxon signed-rank test were used for unmatched and paired samples, respectively, and the Kruskal-Wallis ANOVA using Dunn test for multiple comparisons. Correlations were performed using the nonparametric Spearman rank test. A *P* value <.05 was considered statistically significant.

## RESULTS

We longitudinally analyzed the frequency and activation profile of *M*. *tuberculosis*-specific CD4 T cells in 23 HIV-infected individuals at baseline (day 0 of ART initiation) and at 1, 3, and 6 months of ART, in parallel with the reconstitution profile of the whole CD4 T-cell compartment, compared to a group of 28 HIV-uninfected individuals.

**Figure 1.**
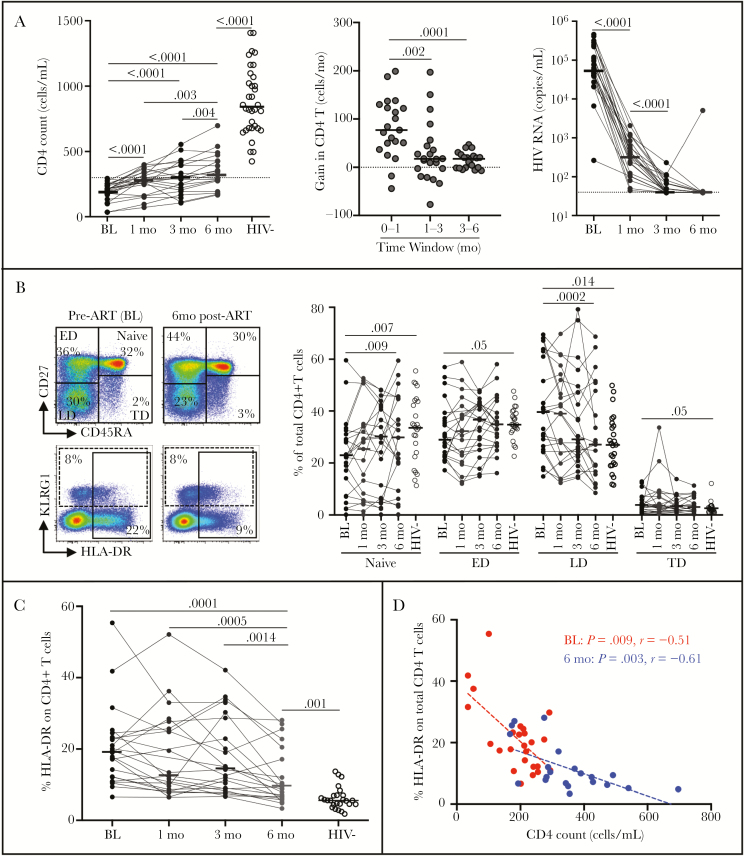
Reconstitution of the CD4 compartment during the first 6 months of antiretroviral therapy (ART) in HIV-1 infected patients (n = 23). *A*, Evolution of absolute CD4 count at baseline (BL, pre-ART), 1, 3, and 6 months’ ART; gain in total CD4 T cells/month; and evolution of plasma HIV viral load. Horizontal lines indicate the median and *P* values are given. *B*, Memory profile of total CD4 T cells from BL to 6 months’ ART. Representative flow plots (left) indicate CD4 T-cell subset distribution and cell activation analysis. Naive cells were defined as CD45RA^+^CD27^+^, early differentiated (ED) as CD45RA^−^CD27^+^, late differentiated (LD) as CD45RA^−^CD27^−^, and terminally differentiated (TD) as CD45RA^+^CD27^−^. *C*, Expression of HLA-DR in total CD4 T cells during ART. Horizontal bars represent the median values. *D*, Relationships between HLA-DR expression in total CD4 T cells and CD4 count at baseline and 6 months’ ART. All statistical comparisons between groups were performed using the nonparametric Mann-Whitney test and correlations were assessed using a 2-tailed nonparametric Spearman rank correlation.

Firstly, we assessed the effect of ART on the whole CD4 compartment. During the first month of ART, the median CD4 count increased significantly compared to baseline (from median 209 to 280 cells/mm^3^). Thereafter, the increase in CD4 count was more modest, reaching medians of 299 and 331 cells/mm^3^ at 3 and 6 months, respectively. This is further illustrated by the fact that the median gain in CD4 T cells/month was significantly higher during the first month of ART (77 cells/month) compared to the following months (approximately 18 cells/month). These changes in absolute CD4 count coincided with successful suppression of HIV viral load, with the median plasma viral load becoming undetectable at 3 months post-ART (Figure 1A).

Next we defined the effect of ART on the memory and activation profile of total CD4 T cells using flow cytometry. CD27 and CD45RA allowed us to discriminate 4 memory subsets (naive, CD45RA^+^CD27^+^; early differentiated, CD45RA^−^CD27^+^; late differentiated, CD45RA^−^CD27^−^; and terminally differentiated, CD45RA^+^CD27^−^). A normalization of the CD4 T-cell memory profile was observed at 6 months of ART, where the median proportion of naive cells significantly increased and late differentiated cells decreased compared to baseline (Figure 1B). KLRG1, a marker associated with cell compartmentalization to vasculature, [11] was measured to define whether HIV impairs CD4 T-cell migration to tissues. We found no difference in KLRG1 expression on total CD4^+^ T cells between the HIV-uninfected and infected groups (median, 11.5% [IQR, 9–21] and 16% [IQR, 8–31], respectively, data not shown). CD4 T-cell activation, as assessed by HLA-DR expression on CD4 T cells, progressively decreased after ART initiation (showing a median 3.4-fold reduction between baseline and 6 months of ART, *P* < .0001). However, CD4 activation after 6 months of ART remained significantly elevated compared to HIV-1 uninfected persons (Figure 1C). Of note, at baseline, 3 and 6 months post-ART, CD4 activation levels correlated with concurrent total CD4 count (Figure 1D and data not shown).

**Figure 2.**
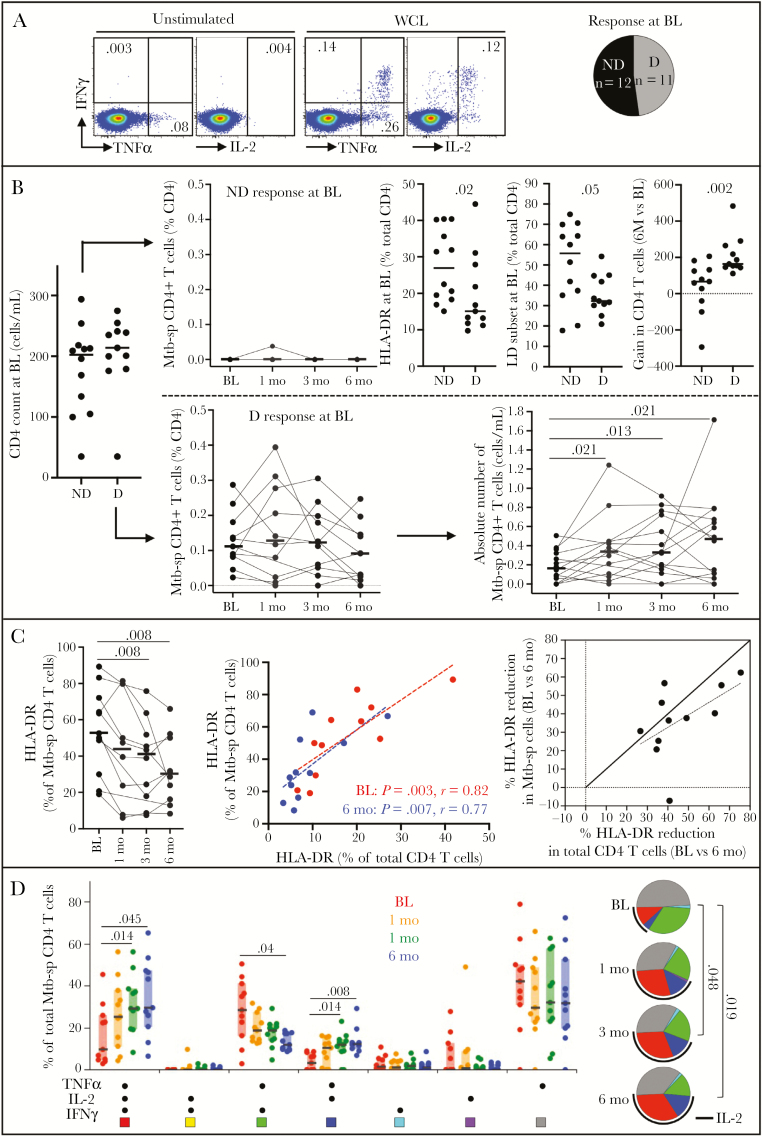
Evolution of Mycobacterium *tuberculosis*-specific CD4 T cells during the first 6 months of antiretroviral therapy (ART) in HIV-1 infected patients (n = 23). *A*, Representative flow plots of interferon-γ (IFN-γ), tumor necrosis factor-α (TNF-α), and interleukin-2 (IL-2) production in response to *M*. *tuberculosis* whole-cells lysate (WCL) and distribution of number of individuals exhibiting a detectable (D) or nondetectable (ND) response to *M*. *tuberculosis* WCL using flow cytometry at baseline (BL). *B*, Evolution of *M*. *tuberculosis*-specific (Mtb-sp) CD4 T-cell responses to ART in individuals with a detectable (D, n = 11) or nondetectable (ND, n = 12) *M*. *tuberculosis* response at BL. Comparison of the activation and memory profile of total CD4 T cells and CD4 cell gain (BL vs 6 months) between individuals with a detectable (D) or nondetectable (ND) *M*. *tuberculosis* response. *C*, HLA-DR profile in *M*. *tuberculosis*-specific CD4 T cells from baseline to 6 months ART and relationship with overall CD4 T-cell activation. *D*, Polyfunctional profile *M*. *tuberculosis*-specific CD4 T cells producing any possible combination of IFN-γ, TNF-α, and IL-2. Each slice of the pie corresponds to a distinct combination of cytokine. A key to colors in the pie charts is shown below the graph and the arc corresponds to the proportion of cells producing IL-2. Horizontal bars and boxes represent the median values and interquartile range, respectively. All statistical comparisons between groups were performed using a nonparametric Mann-Whitney test and correlations were assessed using the 2-tailed nonparametric Spearman rank correlation, and *P* values are given.

We next assessed the evolution of *M*. *tuberculosis*-specific CD4 T-cell responses during ART. At baseline, among the 23 individuals with known *M*. *tuberculosis* sensitization included in this study [9, 10], only 11 participants exhibited a detectable response using flow cytometry (Figure 2A). In participants with no detectable *M*. *tuberculosis*-specific CD4 T cells, ART did not result in a detectable *M*. *tuberculosis*-specific response at any of the follow-up time points. While participants with no measurable response had equivalent CD4 counts compared to *M*. *tuberculosis* responders at baseline, CD4 cells from these patients exhibited elevated expression of HLA-DR and increased memory differentiation compared to *M*. *tuberculosis* responders (*P* = .02 and *P* = .05, respectively). Moreover, they underwent a significantly lower gain in absolute CD4 count over 6 months of ART compared to *M*. *tuberculosis* responders (median, 67 vs 164 cells/mm^3^, *P* = .002) (Figure 2B), suggesting heightened turnover.

In participants with a detectable *M*. *tuberculosis* response, the frequencies of *M*. *tuberculosis*-specific CD4 T cells did not change significantly during the first 6 months of ART. However, due to CD4 T-cell reconstitution, this translates into a significant increase in the absolute number of *M*. *tuberculosis*-specific CD4 T cells as soon as 1 month post-ART initiation (Figure 2B). Qualitatively, the memory maturation profile of *M*. *tuberculosis*-specific CD4 T cells did not change on ART (data not shown), as previously described [7]. However, the activation profile of *M*. *tuberculosis*-specific CD4 T cells decreased, indicated by the significant fall (median, approximately 1.8 fold) of HLA-DR expression on these cells from baseline to 6 months of ART. Notably, the activation profile of *M*. *tuberculosis*-specific CD4 T cells mirrored that of total CD4 T cells both at baseline and 6 months of ART. Additionally, the rate of cell “deactivation” after 6 months of ART was comparable in *M*. *tuberculosis*-specific cells and the overall CD4 compartment (Figure 2C). Finally, the functional potential of *M*. *tuberculosis*-specific CD4 T cells significantly changed as soon as 3 months post-ART, with a significant increase in the proportion of polyfunctional CD4 T cells capable of coproducing TNF-α, IFN-γ, and IL-2, indicating that these *M*. *tuberculosis* antigen-specific T cells regained the ability to produce IL-2 (Figure 2D).

## DISCUSSION

To better understand the dynamics of *M*. *tuberculosis* antigen-specific CD4^+^ T-cell reconstitution during the first 6 month of ART, we first defined the dynamics of replenishment of the whole CD4 T-cell compartment. Our results show that ART induces a rapid expansion of the CD4 compartment over the first month of treatment, postulated to relate to CD4^+^ T-cell redistribution from lymphoid tissues [12], followed by a slower rate of CD4 T-cell recovery after the first month of ART. The normalization of the CD4 memory profile occurs as early as 6 months of ART; however, the activation profile of CD4^+^ T cells was only partially restored, showing that despite complete viral suppression, immune activation persists, as previously reported [13].

The study cohort originated from Khayelitsha township, a highly tuberculosis endemic area, with the prevalence of *M*. *tuberculosis* infection reported to be approximately 69% [8]. When assessing *M*. *tuberculosis*-specific CD4 T-cell responses using flow cytometry, our results show that despite known *M*. *tuberculosis* sensitization, based on IFN-γ release assay and/or ELISPOT assays as described in [9, 10], 12 out of 23 participants had no detectable *M*. *tuberculosis*-specific CD4 T-cell responses. This may reflect the stringent cutoff we applied to define a positive *M*. *tuberculosis*-specific response using flow cytometry, where only *M*. *tuberculosis* responses that were at least 2-fold higher than the background were regarded as positive. In fact, *M*. *tuberculosis*-specific IFN-γ ^+^ CD4 responses pre-ART measured using flow cytometry positively associated with QuantiFERON and ELISPOT results (*P* = .003, *r* = 0.6 and *P* = .0008, *r* = 0.65, respectively, data not shown). It was interesting to note that individuals with undetectable *M*. *tuberculosis* responses exhibited elevated activation and increased memory differentiation in their CD4 compartment pre-ART initiation compared to participants with maintained *M*. *tuberculosis*-specific CD4 T-cell responses. It is thus possible that heightened cell turnover, fueled by HIV-induced systemic immune activation, could have contributed to the depletion of *M*. *tuberculosis*-specific memory CD4 responses to levels undetectable by flow cytometry [14].

During 6 months of ART, in participants with a detectable *M*. *tuberculosis*-specific response pre-ART, the frequency of *M*. *tuberculosis*-specific CD4 T cells remained stable over time even with a significant expansion of the total number of circulating CD4 T cells. This indicates that the restoration of *M*. *tuberculosis*-specific CD4 T cells mirrors the reconstitution of the overall CD4 compartment. Similarly, the “deactivation” of *M*. *tuberculosis*-specific CD4 cells paralleled the change observed in the total CD4 T cells. The functional capacity of *M*. *tuberculosis*-specific CD4 T cells evolved towards a more polyfunctional profile in response to ART, whereby cells regained the ability to produce IL-2. A shift to dominance of CD4 T cells secreting both IFN-γ and IL-2 has been associated with *M*. *tuberculosis* clearance [15]. It is thus plausible that the ART-induced increased proportion of these cells may contribute to *M*. *tuberculosis* protection. However, in individuals without detectable *M*. *tuberculosis* response, 6 months of ART did not result in detectable *M*. *tuberculosis*-specific response. The slower rate of CD4 reconstitution observed in these participants (ie, median of 67 CD4 T cells/mm^3^ over 6 months of ART) may in part explain the lack of restoration of *M*. *tuberculosis*-specific CD4 T cells. As it has been described that *M*. *tuberculosis*-specific CD4 T cells are a preferential target for HIV [3], it is also plausible that chronic HIV infection leads to the complete depletion of *M*. *tuberculosis*-specific memory CD4 T cells, predisposing these individuals to increased risk of active tuberculosis.

While our study has a number of limitations, being a preliminary study with small numbers, the results overall support and reinforce the relevance of generalized recommendation of early ART initiation, to decrease the risk of tuberculosis in HIV-infected individuals, as it may contribute to the maintenance of polyfunctional CD4 T-cell based immune memory responses against *M*. *tuberculosis*.

## Supplementary Data

Supplementary materials are available at *The Journal of Infectious Diseases* online. Consisting of data provided by the authors to benefit the reader, the posted materials are not copyedited and are the sole responsibility of the authors, so questions or comments should be addressed to the corresponding author.

jiz417_suppl_Supplementary_Figure

## Notes

***Author Contributions.*** C. R. and K. A. W. designed the study and drafted the manuscript; N. J. performed the experiments; C. R. and N. J. analyzed the data; M. X. R. contributed to patient recruitment; K. A. W. contributed to sample collection and storage; R. J. W. contributed resources, design, and critical evaluation of the findings. All authors read, critically revised, and approved the final manuscript.

***Financial support.*** This work was supported by The Wellcome Trust (grant numbers 106360, 104803, and 203135); the South African National Research Foundation (grant number 443386), the European Union Horizon 2020 Research and Innovation Programme (grant number 643381; European and Developing Countries Clinical Trials Partnership grants to M. X. R. and TMA2017SF-1951 – TB/SPEC to C. R.); the Medical Research Council of South Africa; The Francis Crick Institute, which receives support from Medical Research Council (MRC) (grant number FC001218), Cancer Research UK (grant number FC001218), and The Wellcome Trust (grant number FC001218); and National Institutes of Health (grant number U19AI111276 to R. J. W. and C. R.).

***Potential conflicts of interest.*** All authors: No reported conflicts of interest. All authors have submitted the ICMJE Form for Disclosure of Potential Conflicts of Interest. Conflicts that the editors consider relevant to the content of the manuscript have been disclosed.
