# Supplementary figures and images for: Tuberculosis Antigen-Specific T-Cell Responses During the First 6 Months of Antiretroviral Treatment
Source: J Infect Dis. 2019 Aug 17;221(1):162–7. doi: 10.1093/infdis/jiz417 (PMC6910879; doi:10.1093/infdis/jiz417)

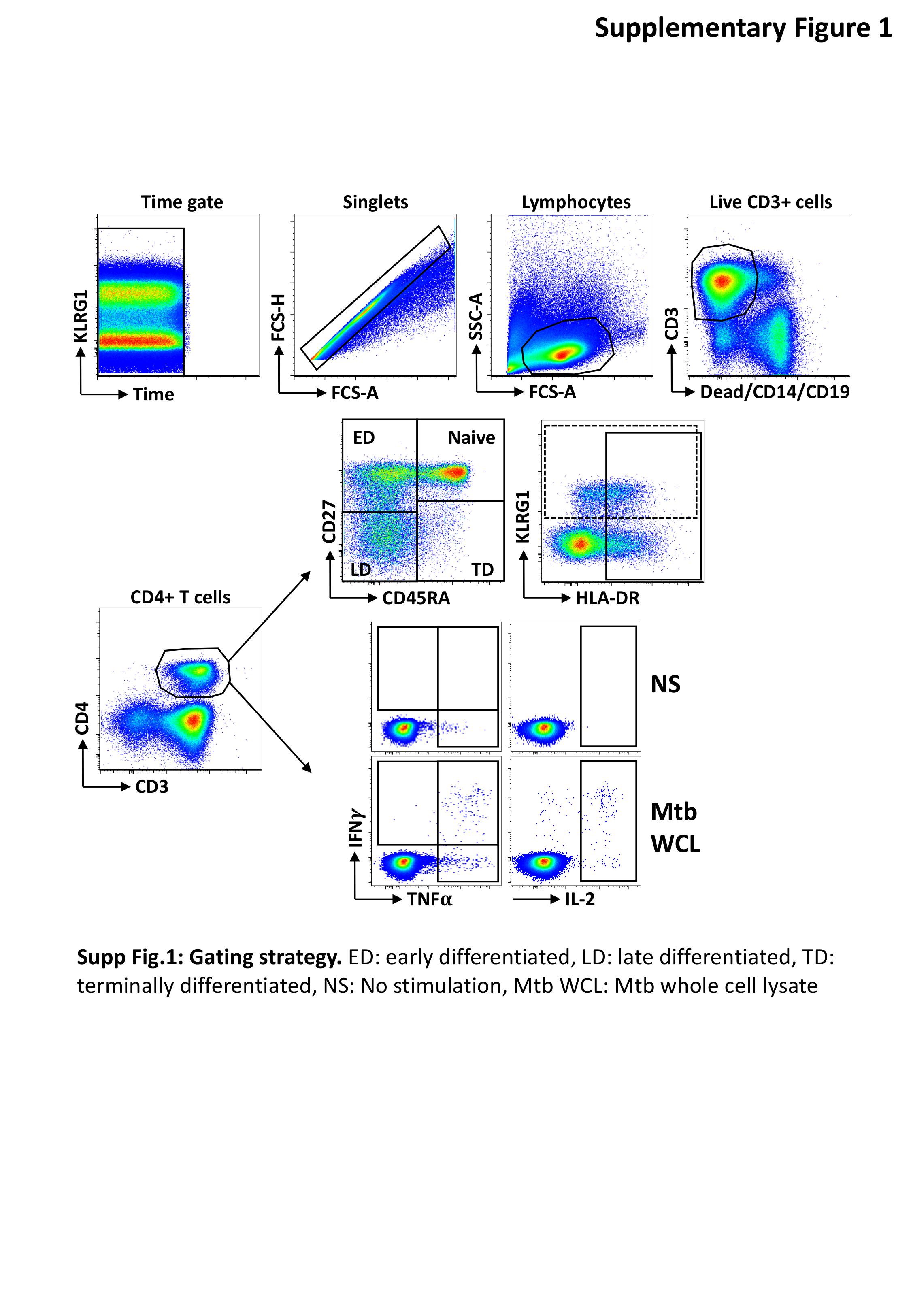

Supplement: jiz417_suppl_Supplementary_Figure [file jiz417_suppl_supplementary_figure.png]
